# World Medical Association Declaration of Geneva

**DOI:** 10.4314/ahs.v17i4.30

**Published:** 2017-12

**Authors:** Anonymous

The Physician's Pledge

Adopted by the 2^nd^ General Assembly of the World Medical Association, Geneva, Switzerland, September 1948 and amended by the 22^nd^ World Medical Assembly, Sydney, Australia, August 1968 and the 35^th^ World Medical Assembly, Venice, Italy, October 1983 and the 46^th^ WMA General Assembly, Stockholm, Sweden, September 1994 and editorially revised by the 170^th^ WMA Council Session, Divonne-les-Bains, France, May 2005 and the 173^rd^ WMA Council Session, Divonne-les-Bains, France, May 2006 and the WMA General Assembly, Chicago, United States, October 2017.

AS A MEMBER OF THE MEDICAL PROFESSION:

I SOLEMNLY PLEDGE to dedicate my life to the service of humanity;

THE HEALTH AND WELL-BEING OF MY PATIENT will be my first consideration;

I WILL RESPECT the autonomy and dignity of my patient;

I WILL MAINTAIN the utmost respect for human life;

I WILL NOT PERMIT considerations of age, disease or disability, creed, ethnic origin, gender, nationality, political affiliation, race, sexual orientation, social standing, or any other factor to intervene between my duty and my patient;

I WILL RESPECT the secrets that are confided in me, even after the patient has died;

I WILL PRACTISE my profession with conscience and dignity and in accordance with good medical practice;

I WILL FOSTER the honour and noble traditions of the medical profession;

I WILL GIVE to my teachers, colleagues, and students the respect and gratitude that is their due;

I WILL SHARE my medical knowledge for the benefit of the patient and the advancement of health care;

I WILL ATTEND TO my own health, well-being, and abilities in order to provide care of the highest standard;

I WILL NOT USE my medical knowledge to violate human rights and civil liberties, even under threat;

I MAKE THESE PROMISES solemnly, freely, and upon my honour.

©2017 World Medical Association Inc. All Rights Reserved. All intellectual property rights in the Declaration of Geneva are vested in the World Medical Association.

